# Monitoring Change in Child Mortality through Household Surveys

**DOI:** 10.1371/journal.pone.0137713

**Published:** 2015-11-25

**Authors:** Kenneth Hill, Eoghan Brady, Linnea Zimmerman, Livia Montana, Romesh Silva, Agbessi Amouzou

**Affiliations:** 1 Institute for International Programs, Department of International Health, The Johns Hopkins University, Baltimore, MD, United States of America; 2 Department of Global Health and Population, Harvard School of Public Health, Boston, MA, United States of America; 3 UNICEF, New York, NY, United States of America; Institute for Health & the Environment, UNITED STATES

## Abstract

**Background:**

Most low- and middle-income countries lack fully functional civil registration systems. Measures of under-five mortality are typically derived from periodic household surveys collecting detailed information from women on births and child deaths. However, such surveys are expensive and are not appropriate for monitoring short-term changes in child mortality. We explored and tested the validity of two new analysis methods for less-expensive summary histories of births and child deaths for such monitoring in five African countries.

**Methods and Findings:**

The first method we explored uses individual-level survey data on births and child deaths to impute full birth histories from an earlier survey onto summary histories from a more recent survey. The second method uses cohort changes between two surveys in the average number of children born and the number of children dead by single year of age to estimate under-five mortality for the inter-survey period. The first method produces acceptable annual estimates of under-five mortality for two out of six applications to available data sets; the second method produced an acceptable estimate in only one of five applications, though none of the applications used ideal data sets.

**Conclusions:**

The methods we tested were not able to produce consistently good quality estimates of annual under-five mortality from summary birth history data. The key problem we identified was not with the methods themselves, but with the underlying quality of the summary birth histories. If summary birth histories are to be included in general household surveys, considerable emphasis must be placed on quality control.

## Introduction

Child mortality in low- and middle-income countries (LMICs) has declined dramatically over the last two decades; the Under-5 Mortality Rate (U5MR) for this group of countries is estimated to have declined by 50% between 1990 and 2013, an annual rate of reduction of 3.0% [1]. These declines have coincided with major investments in child survival programs by governmental and non-governmental agencies, which are keen to monitor the impact of their investments through estimates of change in U5MR for short, recent time periods. Most LMIC countries, however, lack the fully functional civil registration systems required for such rapid monitoring, and estimates of U5MR and other measures of childhood mortality are not derived from continuous recording systems but rather from periodic household surveys, mainly collecting data in the form of full birth histories (FBH). Such surveys, pioneered by the World Fertility Survey [2] in the 1970s and widely implemented since the late 1980s by, among others, the Demographic and Health Surveys (DHS) program [3] are extensively used in the estimation of U5MR in LMICs, but for reasons elaborated below do not lend themselves to rapid monitoring.

In 2008, the Institute for International Programs (IIP) at Johns Hopkins University embarked on a project to test alternative approaches to rapid mortality monitoring (RMM); the overall design of the project and the criteria for selecting countries are described elsewhere in this Series [4]. One type of approach tested was innovative uses of household survey data simpler than the FBH, and we report in this paper on our results.

The FBH asks a representative sample of women about the date of birth, survival status, and (if the child has died) age at death of every one of her live-born children. Such questions are interview time- and interviewer training-intensive, and therefore the cost per interview is high. Additionally, in settings of moderate to low child mortality, particularly where combined with low fertility, large samples of women are required to achieve acceptable sampling errors; thus the FBH methodology is expensive to implement. The periodicity of such surveys is typically five years or more, and adequately precise estimates of U5MR and other indicators cannot be calculated for short time periods [5]. As a result, large annual surveys including FBHs cannot be used for RMM since they would be prohibitively expensive.

Other ways of using birth histories to measure child mortality have been developed. The most widely used is the summary birth history (SBH), whereby women are asked about the number of children they have given birth to and the number of those children that have died. In combination with the age of the woman, or alternatively duration of marriage, or time since first birth, probabilities of dying in childhood can be derived from the proportions dead of children ever born through modeled relationships [6–9]. However, the number of children ever born to women of a given age, and the number of those children that have died, clearly reflect births over a number of years prior to a survey, and deaths of those children over a range of ages. The mortality estimates derived from the proportion of children ever born that are deceased are thus weighted averages of mortality risks over both age and time period, and are neither time-period nor age-range specific, limiting their value for RMM purposes. On the other hand, the SBH questions are much less interview time- and interviewer training-intensive than FBH questions, and therefore much less expensive per interview (so much so that SBH questions are often included in LMIC population censuses).

In light of the cost advantages of the SBH, and therefore the potential to collect information frequently from much larger numbers of women than would be possible with an FBH, the RMM Project was designed to test two new approaches based on SBHs that it was hoped would overcome the two key SBH shortcomings of lack of age range and time period specificity. In practice, cost savings could be even larger, because SBHs would not necessarily need specially-conducted surveys, but could be included opportunistically in other representative household surveys, as shown for example by their inclusion in population censuses. We will refer to the two methods as the Birth History Imputation method (BHI) and the Cohort Change method (CC). The performance of both methods is critically dependent on the quality of the SBHs; methods for evaluating the quality of such histories are fully described elsewhere [9].

## Methods

The BHI method, first explored by Montana [10], is predicated on the observation that almost all LMICs have carried out at least one FBH survey, and on the assumption that the internal dynamics (that is, birth intervals between children and ages at death of children that die) of individual birth histories change fairly slowly as fertility and child mortality change, whereas the distribution of birth histories by numbers of children ever borne (CEB) and those that have died (CD) across women will change more rapidly. Given this assumption, it is possible to improve the specificity of estimates derived from an SBH by borrowing FBHs from an earlier FBH survey for the same population and imputing them onto SBH women. However, the BHI method can never provide true period-specific estimates. Suppose, for example, that child mortality fell to zero in a year; the imputation, using FBHs from an earlier survey, would still impute child deaths into the year, and therefore produce non-zero mortality estimates. The method will, in theory, work best in situations in which child mortality is changing fairly steadily over time, and mortality differentials by, for example, age of mother, or preceding birth interval, are not changing.

The method works as follows. Women in each survey are grouped by five-year age range (or duration of marriage, or time since first birth), number of CEB and number of CD; a woman in a given SBH category (for example, age 25 to 29, 3 CEB, 1 CD) will be assigned, at random, an FBH from a woman in the same category in the FBH. To reduce random variation, the matching is repeated 10 times; the matching is carried out with replacement, so if there are fewer cases in the FBH dataset than in the SBH dataset, the FBH dataset is expanded by the required factor prior to matching. The SBH with the appended FBHs is then analyzed by time period and age range as if it were collected as an FBH. The explicit assumption made by the method is that, for women in a given category, the time distribution of births and age distribution of child deaths do not change over time. In practice, the effect of this assumption is mitigated by geographic and temporal proximity: using FBHs from the same (or a neighboring) country, and for a time point as close to the date of the SBH as possible.

The BHI method is entirely straightforward to implement, except in one respect: how to treat non-matches, that is, cases in the SBH that do not have matches in the FBH (cases in the FBH that do not have matches in the SBH are irrelevant). We tested two approaches: first, reducing the number of matching categories to CEB and CD only, dropping the woman’s age, and then dropping any small number of cases that still remain unmatched; and second, creating from all available DHS FBH surveys a compendium of all recorded category combinations, each with up to 10 FBH, and then borrowing from this master set for all unmatched cases; the very small number of cases still unmatched are then dropped. The compendium method performed much better in terms of proportions ultimately matched and approximating the proportion of deceased children from those ever born by age of the mother (the key measure for standard indirect estimation) in the original data set, so we only present results based on the compendium method here.

The BHI method was tested in all five RMM Project countries, in one case more than once. In principle, the validation approach followed was to take a nationally-representative SBH, impute onto it an FBH from an earlier DHS FBH survey, compute child mortality indicators (neonatal, infant and under-five mortality) from the imputed FBH for 10 calendar years before the (SBH) survey, and then compare these estimates with those computed for the same calendar years from a subsequent DHS or other FBH survey. In practice, however, the applications were in all cases but one made to sub-national populations, as a consequence of the design of the overall RMM Project. Clearly, estimates from the validation survey will be affected by sampling errors (and possibly by systematic errors as well), but estimates from such surveys are regarded as current best practice in countries lacking full civil registration.

**Fig 1:**
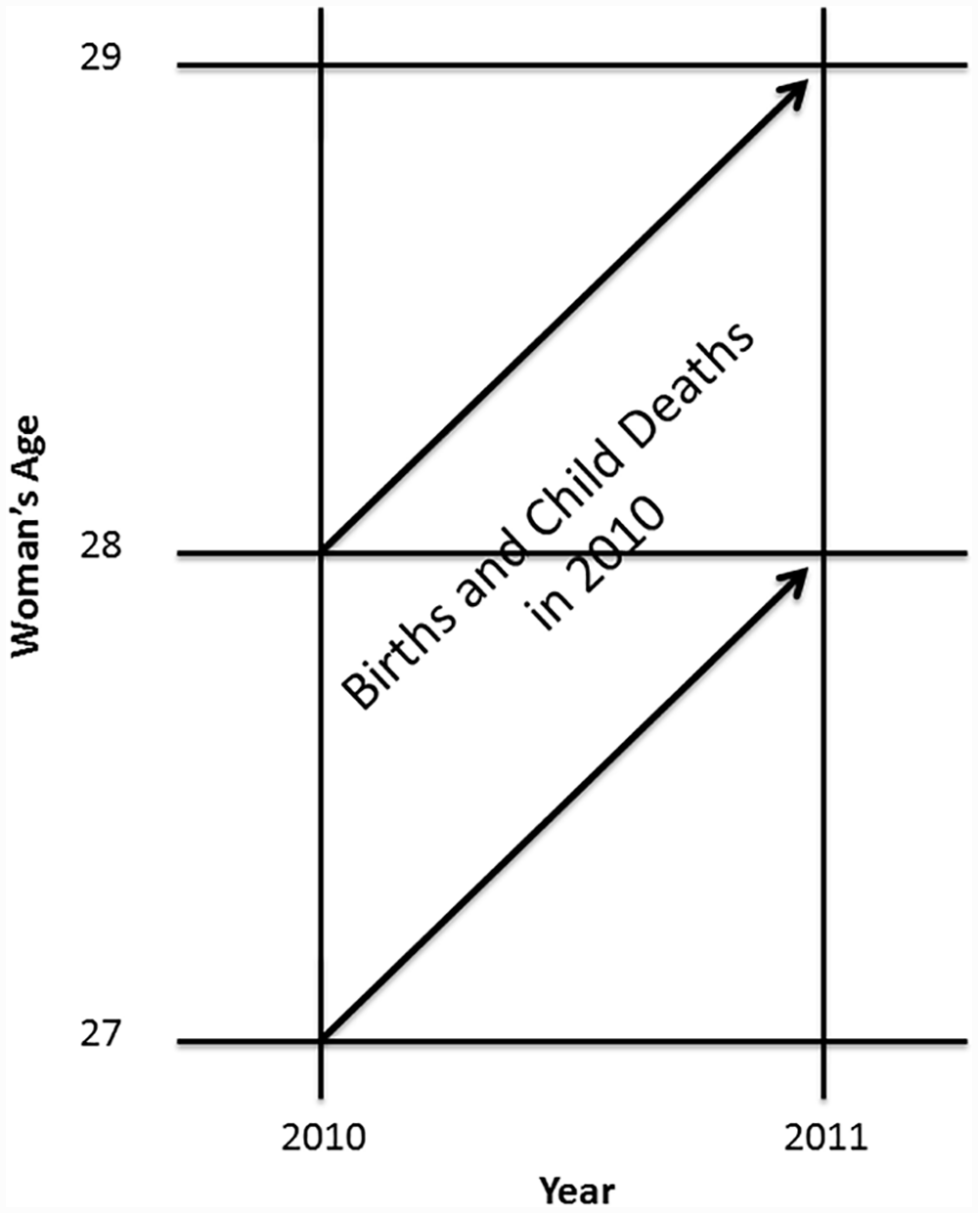
The Intersurvey Cohort Change Method. This figure illustrates the Cohort Change Method schematically.

The CC method is an extension of a method proposed by Zlotnik and Hill [11], and primarily addresses the issue of a lack of time specificity in SBH estimates; it does not produce estimates that are specific by age of child. The key assumption of the method is that repeated survey cross-sections can be treated as quasi-cohorts; for example, that women aged 27 at representative survey one held at the end of 2010 and women aged 28 at representative survey two at the end of 2011 can be regarded as being a cohort of women born in 1983. Other things being equal, change in average CEB for each cohort from one year to the next is driven by the fertility rate for the cohort during the year, and change in CD is driven by child deaths (at any age) during the year. We illustrate the basic concept (Fig 1). Changes in average CEB and average CD from one survey to the next for all quasi-cohorts of women of reproductive age can then be cumulated, and the sums will reflect fertility (the sum of the changes in CEB will be equal to the total fertility rate for that period) and child mortality during the year, but will be unaffected by births and deaths before the start of the year. The ratio of cumulated CD change (c-CD) to cumulated CEB change (c-CEB) will largely be determined by the risk of under-five mortality during the year.

**Fig 2:**
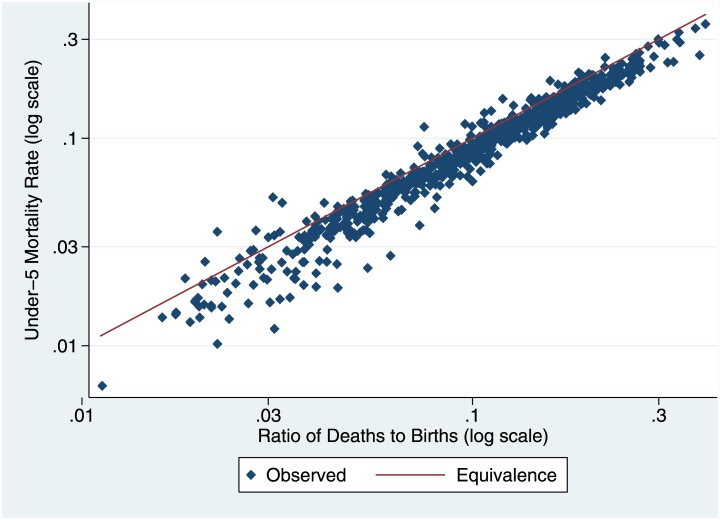
Relationship between ratios of cumulated cohort changes in CD to CEB and U5MR in DHS: One Year Period (log scale). This figure shows the relationship between annual estimates of U5MR and ratios of births to deaths for one- or two-year periods from 168 Demographic and Health Surveys.

**Fig 3:**
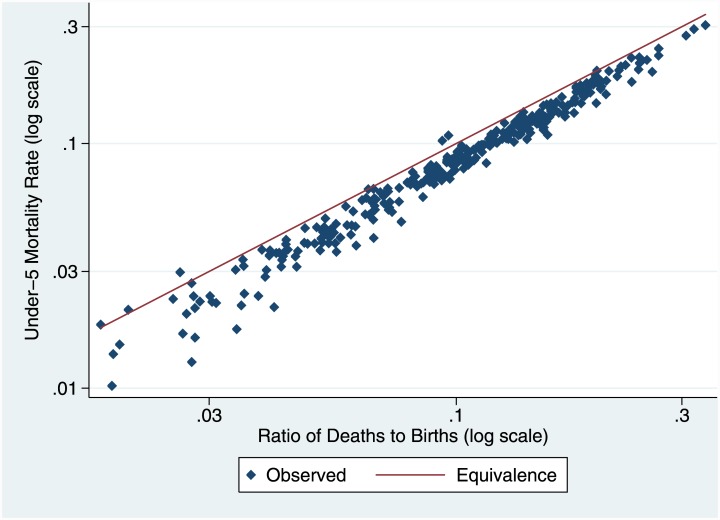
Relationship between ratios of cumulated cohort changes in CD to CEB and U5MR in DHS: Two Year Period (log scale).

To explore the relationship between the c-CD/c-CEB ratio and standard measures of child mortality, we extracted data from 154 DHS surveys covering 69 LMICs on numbers of births and deaths (at any age) of children for each of the five calendar years before each survey, and also calculated period measures of infant and child mortality using standard methods [8] for the same five years. We show the relationship between the natural log of the c-CD/c-CEB ratio and the natural log of the under-five mortality rate (U5MR) in Fig 2. The relationship is remarkably close; as a first-order approximation, the ratio for a particular year measures U5MR for that year. We show the relationship for two-year as opposed to one-year periods in Fig 3; again, correspondence is close. However, since our outcome of interest is U5MR, the exact relationship will be affected by factors that affect the age distribution of children’s deaths. Consider, for example, a case in which fertility has been falling. The age distribution of surviving children (to women of all ages) will be older (than in a constant fertility case), reflecting the births in the past when fertility was higher, and the age distribution of child deaths will also be correspondingly older; the ratio will then over-estimate U5MR, because the age distribution of deaths will be shifted from below five to above five. Similarly, a high proportion of women of older reproductive age (for example, 40 to 44), who have largely completed their childbearing, relative to younger women, will tend to increase c-CD as a result of deaths of their children at ages over five relative to those under age five; an age pattern of child mortality with high mortality above age five relative to that under age five, that might result for example from a generalized HIV epidemic, will have the same effect. We use the same data set from DHS to develop a regression-based correction method using only indicators collected in an SBH or available from external sources.

An additional complication arises from the common error in surveys of low- and middle-income countries of age heaping—that is, differential reporting of ages ending in digits 0 and 5. This error would not be a problem if it were not also associated with reported values of CEB and CD, but in practice there is often a sharp step up or down in average CEB and average CD between one single year of age and the next; such a pattern would arise for example if women who could not accurately report their age have higher fertility and child mortality risks than women who could. Since the reported size of cohorts ending in 0 and 5 is larger than that of neighboring cohorts, the effects of cohort changes between one survey and the next will be exaggerated or diminished. To reduce the impact of this error, we use five year cohorts incremented by single years of age, thus computing cohort change for 15–19 (16–20, 17–21, etc.) year olds at the first survey, and 16–20 (17–21, 18–22, etc.) year olds at the second.

For estimation purposes, we use the following model:

$$\text{ln}\left(U5MR_{t}\right)=\beta_{0}+\beta_{1}\text{ln}\left(\frac{c-CD_{t}}{c-CEB_{t}}\right)+\beta_{i}X_{it}$$ (1)

where the *X*_*it*_ are variables controlling for factors likely to influence the age distribution of deaths of children in year *t*. In practice, we use the ratio of the female population aged 20–24 to that aged 40–44 to control for female age distribution, HIV prevalence five years before the observation as estimated by UNAIDS [12] as one control for age pattern of mortality of children, total fertility (c-CEB) in year *t* to cohort lifetime fertility (average parity of women aged 40–49) to control for fertility change, and the ratio of the proportion deceased of CEB for women aged 25–29 to that for women aged 45–49 as an additional control for the age pattern of child mortality.

### Ethical review

We obtained approval from the Johns Hopkins Bloomberg School of Public Health ethical review board for the studies that involved primary data collection (Ethiopia [13] and Mali [14] endline surveys, Ethiopia baseline survey [13], and Malawi midline survey [15]; survey methodology is described in the referenced papers). For those studies, participants provided oral consent. The ethical review board explicitly approved the method for obtaining verbal consent, which was recorded by the interviewer; for persons under the age of 18, parent/guardian consent was obtained in the same way. We also used previously published data from the DHS (available at www.dhsprogram.com/data) and MICS (available from www.mics.unicef.org/surveys) survey programs.

## Results

We first describe results of the methodological development of the cohort change method based on DHS FBH data, and then provide results of applications of both the imputation and the cohort change methods to data from the five RMM countries.

All datasets collected through the RMM project are open access and can be found at http://dx.doi.org/10.7281/T1F769G3, along with their associated documentation. Additional survey datasets used for the calibration, testing, and validation of the RMM rapid survey methods are available through the ICF Macro and UNICEF portals associated with the Demographic and Health Survey program and the Multiple Indicator Cluster Surveys program [16,17].

The methodological issue for cohort change is the relationship between the c-CD/c-CEB ratio and U5MR. Table 1 shows results of regressions using the formulation in Eq (1). Results are presented for survey intervals of both one year and two years. The first and third columns show the regressions without controls, the second and fourth with controls. The simple relationship between ln(U5MR) and ln(c-CD/c-CEB) is close to one to one (Figs 1 and 2), with intercepts close to 0 and slopes only slightly greater than one; the simple relationship accounts for over 95% of the variance in ln(U5MR). Three of the control variables (female age distribution, fertility change, and ratio of proportions dead) were highly significant (p<0.001), and the fourth (lagged HIV prevalence) was significant at 5%. Adding the controls improves the fit somewhat (over 96% of variance); interestingly, the coefficient on the main dependent variable becomes insignificantly different from 1.0.

**Table 1:** Regression Coefficients for Relationship between Ratios of Cumulated Cohort Changes in CD to CEB and U5MR in DHS.

*Dependent Variable*: *ln(U5MR*_*t*_ *)*				
	One-Year Intersurvey Interval		Two-Year Intersurvey Interval	
ln(c-CD/c-CEB)_t_	1.041 (1.025,1.056)	0.981 (0.961,1.000)	1.062 (1.043,1.081)	0.996 (0.973,1.020)
Female Population 20-24/Female Population 40–44		0.066 (0.041,0.090)		0.068 (0.040,0.097)
Female HIV Prevalence, 5 Years before survey		-0.0026 (-0.0046, -0.0005)		-0.0033 (-0.0056, -0.0010)
TFR/Cohort Lifetime Fertility, Year *t*		0.364 (0.276,0.452)		0.313 (0.199,0.426)
Prop. Dead (25–29)/Prop. Dead(45–49) (survey)		0.183 (0.091,0.274)		0.170 (0.066,0.274)
Intercept	-0.050 (-0.088,-0.012)	-0.721 (-0.840,-0.602)	-0.034 (-0.080,0.012)	-0.667 (-0.816,-0.518)
R^2^	0.955	0.966	0.974	0.981
N of observations	793	708	316	280

95% CI in parentheses. Results are drawn from 154 DHSs covering 69 countries.

We now turn from results of methodological development to application and validation. As described above, validation consists of comparing annual estimates of mortality indicators derived from an imputed full birth history or from cumulated cohort change to estimates for the same calendar year or time period drawn from a later full birth history. The values against which we validate are thus only as good as the surveys from which they are taken, and are potentially affected by both sampling and non-sampling errors; however, the full birth history is regarded as the current best practice for measuring child mortality in countries lacking accurate vital statistics, and we use the same approach for validating other RMM methods tested.

**Fig 4:**
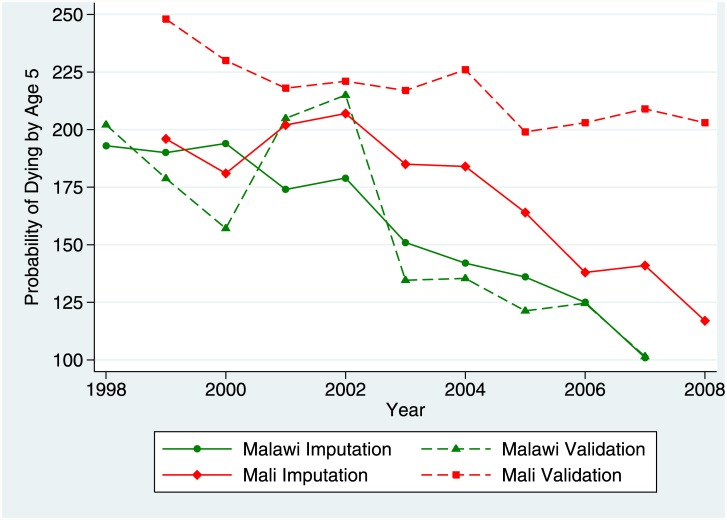
Annual estimates of U5MR from imputation and validation data sets: Malawi and Mali. This figure shows annual estimates of U5MR from the Birth History Imputation Method on the one hand and from the validation survey on the other for applications to Malawi and Mali.

The BHI method has been applied to data from six surveys across the five study countries (two applications were made to data from Ethiopia). In all cases except one (Niger), the FBHs used for imputation were derived from a prior DHS, and applied to a subsequent survey with only an SBH, the SBH having been collected by a population census in four cases and by a project survey in one case; in all these cases, the study population was sub-national. In Niger, there has been no large-scale SBH survey, so the imputation method was applied to nationally-representative data by treating the 2010 Mortality Survey (ESM), which collected an FBH, as if it were an SBH, and imputing full histories onto it from the 2006 DHS. Table 2 summarizes the results of the applications in terms of estimates of under-five mortality rates; we illustrate results for two applications, for Malawi and Mali, in Fig 4, which compares imputed and validation U5MR estimates for single calendar years. In two out of six applications, overall accuracy appears reasonably good, with mean relative error (computed as {BHI estimate—validation estimate}/validation estimate) of 2% or less; errors tend to be larger for neonatal and infant mortality (not shown). However, in two applications, mean relative error in U5MR is a 10% or greater underestimate, and in the remaining two applications the mean relative error is greater than a 10% overestimate. Also, even in the two successful applications, the small mean relative error results from a combination of under-and over-estimates cancelling each other out, such that the mean absolute relative errors are between 7% and 10%. Another useful validation metric is the number of annual estimates (out of 10) for which the relative error was greater than 20%; for two applications (Malawi and Niger) there were one or fewer such cases, and in one application (Ghana) there were only two such cases, both over-estimates For the remaining three applications, four or five of the annual estimates were off by 20% or more—in two cases all under-estimates, and in one case all over-estimates.

**Table 2:** Summary of Average Accuracy and Variability of Birth History Imputation Annual Estimates of Under-Five Mortality Rates Relative to Validation Estimates.

Country	Data Sources			Accuracy		Variability: Number of Years (out of 10) With:		
	Imputation FBH	SBH	Validation FBH	Mean Relative Error^a^	Mean Absolute Relative Error^b^	Low Estimates^c^	Acceptable Estimates^d^	High Estimates^e^
Ethiopia^f^	DHS 2005 (1,545)	Census 2007 (11,725)	Endline Survey 2013 (19784)	-0.12	0.13	4	6	0
Ethiopia^f^	DHS 2011 (1,469)	Baseline Survey 2011 (10,925)	Endline Survey 2013 (19,784)	0.19	0.24	0	6	4
Ghana^g^	DHS 2008 (2,401)	Census 2010 (35,808)	MICS 2011 (6,610)	0.11	0.14	0	8	2
Malawi^h^	DHS 2004 (8,130)	Census 2008 (11,765)	DHS 2010 (1,293)	0.02	0.10	0	9	1
Mali^i^	DHS 2006 (1,373)	Census 2009 (84,737)	Endline Survey 2013 (4,820)	-0.21	0.21	5	5	0
Niger^j^	DHS 2006 (7,205)	ESM 2010 (21,826)	DHS 2012 (9,209)	0.00	0.07	0	10	0

*Notes*:

Numbers in parentheses under each survey are number of women 15–49 interviewed.

^a^ Mean relative error calculated as (U5MR_BHI_-U5MR_Validation_)/ U5MR_Validation._

^b^ Mean absolute relative error calculated as (|U5MR_BHI_-U5MR_Validation_|)/ U5MR_Validation._

^c^ Estimates are classified as low if they are less than 80% of the validation estimate.

^d^ Estimates are classified as acceptable if they are within 20% of the validation estimate.

^e^ Estimates are classified as high if they are greater than 120% of the validation estimate.

^f^ Study areas of Jimma and West Hararghe Districts for SBH and FBH validation data, Oromia region for imputation FBH.

^g^ Northern Region SBH data, FBH data for imputation and validation Ghana rural areas.

^h^ Study districts of Salima and Balaka for SBH and FBH validation data, Malawi rural areas used for FBH imputation data.

^i^ Study districts of Niono and Barouelli for SBH and FBH validation data, Segou region used for FBH imputation data.

^j^ National.

There are a number of potential sources of error in the BHI method, but the most basic source of difference from a validation data set may have nothing to do with the method, but may arise from the quality of the summary birth history itself; if proportions of deceased children are very different in the two data sets, the U5MR estimates will also be very different. The fact that all the large (>20%) relative errors for a particular data set were in the same direction supports the explanation that it is the SBH (or validation FBH) that is at fault. Table 3 compares proportions deceased of children ever born by age group of mother from the SBH survey and the validation survey for all six applications. For the two relatively successful applications (Malawi and Niger), these proportions are relatively similar between the imputed and validation data sets, whereas for the Mali application the SBH proportions derived from the census are much lower than those from the validation data set. In the Ghana application and the second Ethiopia application, the proportions of deceased are higher in the SBH survey than in the validation survey, giving rise to an upward bias in the BHI estimates.

**Table 3:** Proportions Deceased of Children Born Reported by All BHI and Comparison Surveys.

Age Group of Mothers	Ethiopia			Mali		Malawi		Niger		Ghana	
	2007 Census	2011 Baseline	2013 Endline Survey	2009 Census	2013 Endline Survey	2008 Census	2010 DHS	2010 ESM	2012 DHS	2010 Census	2011 MICS
15–19	0.073	0.104	0.080	0.165	0.173	0.100	0.112	0.115	0.078	0.121	0.060
20–24	0.076	0.106	0.070	0.155	0.195	0.107	0.078	0.127	0.108	0.100	0.083
2529	0.103	0.127	0.093	0.166	0.213	0.143	0.133	0.156	0.154	0.106	0.081
30–34	0.141	0.137	0.112	0.177	0.226	0.179	0.174	0.184	0.177	0.113	0.093
35–39	0.161	0.181	0.131	0.187	0.247	0.200	0.178	0.213	0.217	0.128	0.113
40–44	0.213	0.230	0.165	0.205	0.270	0.231	0.209	0.243	0.256	0.155	0.136
45–49	0.216	0.255	0.196	0.217	0.284	0.270	0.266	0.263	0.282	0.165	0.147

*Note*: Geographical representation is as indicated in Table 2.

Another potential source of error in the BHI method is the treatment of first step non-matches. As described in the Methods section, we found the approach of matching those cases not matched on the first round to a compendium of CEB/CD/Age of mother types drawn from all DHS surveys to be superior. We therefore report only on the second (compendium) method. Table 4 shows, by application, the proportions of all cases that matched on the first step, and the additional proportions that ultimately matched on the second step. In general, the proportion of first step matches is high, 93% to 98%, and the additional matches from the compendium range from 1.2% to 6.7%. The final proportion of non-matches is extremely low in all cases except Mali, where 0.6% of cases never matched. Further exploration of the somewhat lower match rate for Mali indicates that it was largely due to some implausibly extreme CEB/CD values for women under age 25 in the 2009 census.

**Table 4:** Birth History Imputation Method: Proportions of Cases Matched.

Application	First stage match	Second stage match: Compendium	Percentage unmatched
Ethiopia (Census 2007)	96.1%	3.9%	0.0%
Ethiopia (Baseline 2011)	94.2%	5.8%	0.1%
Ghana	95.3%	4.7%	0.0%
Malawi	98.0%	2.0%	0.0%
Mali	92.8%	6.7%	0.6%
Niger	98.7%	1.2%	0.1%

*Note*: Geographical representation is as indicated in Table 2.

We applied the cohort change (CC) method twice in three of the five RMM countries, for a total of six applications. Table 5 shows the data sources used and summarizes the results. We developed the CC method to be applied to successive SBH surveys that use comparable data collection methodology and are separated by one or two years, but none of our applications meet this ideal. In four cases, we applied the method to a pair of surveys, one of which was an SBH survey, and one an FBH survey; in the other two cases we applied the method to a pair of FBH surveys. We expected the cohort changes in CEB and CD to be sensitive to data collection procedures, hence the need for comparable methodology, so applying the method to data from surveys using different types of data collection was less than ideal. Even given this caveat, however, the results were extremely disappointing. In only one of the six applications was the cumulated cohort change in average numbers of deceased children a positive number (a negative number suggests under-reporting of dead children at the second survey relative to the first). For what it is worth, in the one application (Malawi) for which the cumulated cohort change in CD was positive, the estimated U5MR was close (a 6% underestimate) to the validation estimate for the same time period.

**Table 5:** Results of Cohort Change Method.

Country	Data Sources			Cumulated Cohort Change		Estimated U5MR (‘000)	
	*First Survey*	*Second Survey*	*Validation*	*CEB*	*CD*	*Cohort Change*	*Validation*
Ethiopia^a^	DHS 2005 (14,070)	Census 2007 (1,737,461)	DHS 2011 (16,515)	-0.86	-2.96	*	75.1 for 2006
Ethiopia^b^	Baseline 2011 (10,936)	Endline 2013 (26,777)	Endline 2013 (26,777)	2.27	-3.67	**	68.9 for 2012
Ghana^c^	MICS 2006 (5,890)	MMS 2007 (10,370)	MICS 2011 (6,610)	5.09	-0.01	**	93.1 for 2006
Ghana^c^	MMS 2007 (10,370)	DHS 2008 (4,916)	MICS 2011 (6,610)	0.15	-0.22	**	75.8 for 2007
Malawi^d^	Census 2008 (309,851)	DHS 2010 (23,020)	DHS 2010 (23,020)	13.58	1.50	102	109.0 for 2009
Malawi^e^	DHS 2010 (1,635)	Midline “current best practice” 2011 (21,768)	Midline “current best practice” 2011 (21,768)	3.78	-1.14	**	102.8 for 2010

*Notes*:

Numbers in parentheses under each survey are number of women 15–49 interviewed.

*: Cumulated increments of both CEB and CD negative, no estimate possible.

**: Cumulated increments of CD negative, no estimate possible.

^a^ National.

^b^ Study regions of Jimma and West Haraghe.

^c^ National.

^d^ National.

^e^ Study districts of Salima and Balaka.

**Fig 5:**
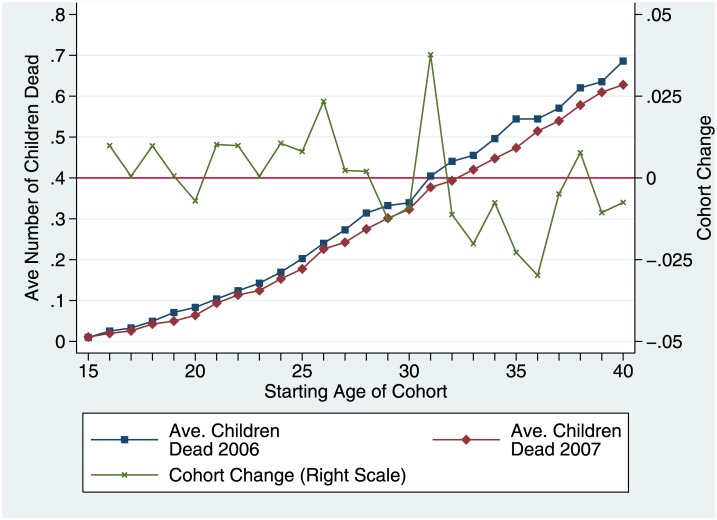
Average numbers of children dead by rolling 5-year cohorts: Ghana 2006 MICS and 2007 MMS. This figure shows the average number of children dead reported by women in rolling five year age groups from 15–19 to 40–44 from two surveys in Ghana separated by one year, and the changes by cohort from one survey to the next.

We use an example from Ghana to illustrate the problem. The initial SBH comes from the 2006 Multiple Indicator Cluster Survey, the second from FBHs from the 2007 Maternal Mortality Survey. We show the average number of deceased children by rolling five-year cohorts of women incrementing by one year of age from 15–19 to 40–44 from each source, and the increments in average number of children dead calculated from one survey to the next for each cohort in Fig 5. The patterns of average numbers of children deceased by age are very similar; age by age the 2006 numbers are higher than the 2007 numbers, as could be the case as a result of declining child mortality. However, the cohort increments from one survey to the next after age 28 are all negative except for two—something which cannot be explained by declining child mortality—and the sum of cohort changes is also slightly negative, by one-hundredth of a dead child (Table 5).

## Discussion

We have developed, and tested the validity of, two methods for analyzing SBH data to obtain more timely or detailed estimates of under-five mortality than standard indirect methods provide. More effective use of such data is attractive because SBHs are relatively inexpensive to collect, thus potentially allowing larger samples and more frequent implementation. SBHs can also be included opportunistically in any representative household survey at modest marginal cost. If SBHs could be used in ways to get more timely and detailed estimates, it would thus be possible to achieve the objective of RMM and monitor and evaluate child survival programs over short periods of time, and also to identify underperforming areas for management purposes.

Unfortunately, our results have been disappointing. The birth history imputation method, which essentially borrows FBHs from an earlier survey and imputes them onto an SBH dataset, gave satisfactory results in only two of six applications. The primary problem, however, was not with the method, but with the quality of the SBHs themselves. Estimates can be no better than the quality of the underlying SBHs, and the quality of the histories, as indicated by proportions deceased of children ever born, varies surprisingly widely from one survey to another. In the two applications (Niger and Malawi) in which the proportions deceased by age of mother in the SBH survey and the FBH survey used for validation were similar, the annual estimates obtained by BHI were also similar to those from the validation data set, so with high quality SBH data, the method can work. Even with perfect data, however, the imputation method does not give true period-specific estimates of childhood mortality; the best it can do is control the resulting mortality estimates for observed changes in the numbers of children born and the numbers that have died to women classified by age (or duration of marriage or time since first birth).

The cohort change method does, in principle, provide a true period-specific estimate of the U5MR, though it does not have the capacity to estimate mortality for different age ranges of childhood. Our applications of the method did not produce satisfactory results, primarily because of the finding that SBHs collected by successive surveys do not produce mutually consistent CEB or (particularly) CD series. A major factor contributing to the inconsistencies is the difference in data collection methodologies between the first and the second surveys; in no case in this study were we able to test an application in which SBH data from highly similar successive surveys were available. It is thus possible that the method could still work in situations in which highly similar SBH surveys with large sample sizes can be repeated every year or so. The method does, in principle, provide period-specific estimates, and the marginal cost of including an SBH in a survey conducted primarily for other purposes should be small. If the problem of consistency of data collection procedures can be resolved, CC may prove to be a satisfactory RMM method. However, given that this study, to our surprise, found substantial variability in SBH quality between surveys, it is unlikely in our judgement that the CC method will be able to provide the consistency of estimates needed for the rapid evaluation of health programs.
